# Chronic adversity and cerebellar structure: Links with neighborhood disadvantage and childhood maltreatment in adult trauma survivors

**DOI:** 10.1016/j.ynstr.2026.100861

**Published:** 2026-11-02

**Authors:** Jamie Terner, E. Kate Webb, Terri A. deRoon-Cassini, Christine L. Larson, Ashley A. Huggins

**Affiliations:** a Department of Psychology, University of Arizona, Tucson, AZ, USA; b Department of Psychiatry and Behavioral Sciences, Duke University School of Medicine, Durham, NC, USA; c Division of Trauma & Acute Care Surgery, Comprehensive Injury Center, Department of Surgery, Medical College of Wisconsin, Wisconsin, USA; d Department of Psychology, University of Wisconsin-Milwaukee, Milwaukee, WI, USA

**Keywords:** Cerebellum, Adversity, Trauma, Neuroimaging, PTSD, Neighborhood disadvantage

## Abstract

Chronic adversity, including neighborhood socioeconomic disadvantage and childhood maltreatment, has been linked to alterations in brain structure and increased risk for psychopathology. While prior research has focused largely on cortical and limbic regions, the cerebellum is increasingly recognized for its role in cognitive and affective processes and may be particularly sensitive to prolonged stress. The present study examined associations between chronic adversity and cerebellar volume in a sample of trauma-exposed adults. Participants (N = 220) were recruited following acute traumatic injury and completed structural magnetic resonance imaging (MRI) post-trauma, along with assessments of neighborhood disadvantage (Area Deprivation Index) and childhood maltreatment (Childhood Trauma Questionnaire). Greater neighborhood disadvantage was significantly associated with smaller total cerebellar volume after adjusting for age, gender, and intracranial volume (*p* < .001). Greater childhood maltreatment was also associated with smaller total cerebellar volume (*p* = .043), although effects were more modest. Exploratory analyses indicated that neighborhood disadvantage was associated with widespread reductions across multiple cerebellar subregions, whereas childhood maltreatment showed more limited and attenuated associations after covariate adjustment. Mediation analyses revealed that total cerebellar volume did not significantly mediate associations between adversity and PTSD symptom severity. These findings suggest that both environmental and interpersonal forms of chronic adversity are associated with reduced cerebellar volume in adulthood. The cerebellum may represent a neural substrate through which chronic adversity becomes biologically embedded.

## 1 Introduction

Chronic adversity encompasses stressors that occur over an extended period, such as growing up in poverty, which often contributes to lasting negative impacts on an individual. Specifically, exposure to chronic adversity is linked to various psychiatric disorders and poor mental and physical health outcomes across the lifespan (Benjet et al., 2010). Ongoing exposure to both personal and environmental stressors, such as trauma and poverty, can have enduring consequences for brain development and functioning. Growing evidence suggests that cumulative exposure to stressors across development exerts a profound impact on neurobiological systems implicated in emotion regulation, cognition, and psychopathology risk (Webb et al., 2021a, Webb et al., 2021b; Suarez et al., 2024; McLaughlin et al., 2019; Schafer et al., 2022).

Socioeconomic disadvantage represents one critical form of chronic adversity. Neighborhood-level indices, such as the Area Deprivation Index (ADI; Kind and Buckingham, 2018), capture structural inequities in income, education, housing, and employment, and have been linked to a broad range of health disparities, including in psychiatric risk and altered brain structure and function (Huggins et al., 2022; Tomas et al., 2022; Webb et al., 2021; Gard et al., 2020). Importantly, neighborhood disadvantage reflects not only economic hardship, but also contextual stress exposures, including reduced access to resources and increased exposure to violence or instability, that can accumulate across the lifespan (Ross and Mirowsky, 2001).

Whereas neighborhood socioeconomic disadvantage reflects a chronic, structural context characterized by sustained deprivation and environmental stressors that accumulate over time, childhood trauma involves direct, often interpersonal experiences of actual or threatened harm that occur during sensitive periods of development. Childhood maltreatment, which includes trauma, can be broadly defined as exposure to experiences of abuse and/or neglect in physical, sexual, or emotional domains during childhood or adolescence. Childhood maltreatment can thus be viewed as another potent form of chronic adversity with well-documented associations to altered neurodevelopment and heightened vulnerability to psychiatric disorders (Rakesh et al., 2021; Nemeroff, 2004). Childhood maltreatment during early sensitive developmental windows can have lasting effects on brain morphology and connectivity, with evidence implicating alterations in regions supporting stress regulation, executive functioning, and affective processing (Callagan and Tottenham, 2016; Maccari et al., 2014; Malave et al., 2022; Schafer et al., 2022).

Although socioeconomic disadvantage and childhood maltreatment differ in their mechanisms and sources of stress, both represent forms of chronic adversity that have been linked to alterations in brain structure and function. Specifically, much research has emphasized the associations between these types of adversity and cortical and limbic structures, including the hippocampus and the amygdala (Kılıç et al., 2024; Assari, 2020). These findings have also been reflected in animal models of chronic stress and the brain (Paquola et al., 2016). Additionally, studies examining whole-brain and regional gray matter volume correlations with adversity have extended these findings into the thalamus and insular cortex (Gehred et al., 2021). Until more recently, the cerebellum was not considered in these analyses. Known for its role in coordinating smooth movement and balance, the cerebellum is increasingly being recognized as a critical hub for integrating cognitive, affective, and sensorimotor processes (Stoodley and Schmahmann, 2010; Moreno-Rius, 2018). Limited functional neuroimaging studies demonstrate topographic organization of the cerebellum, such that sensorimotor functions are primarily localized to the anterior lobe, whereas cognitive and affective processes more consistently engage posterior cerebellar regions including crus I/II, lobules VI–IX, and vermal regions (Stoodley and Schmahmann, 2018). The cerebellum undergoes protracted neurodevelopment, extending into adolescence and early adulthood (King et al., 2019; Borges et al., 2022), suggesting that it may be particularly sensitive to the cumulative and chronic effects of environmental adversity. Cerebellar abnormalities have been observed in trauma-exposed populations and linked to the development of psychiatric symptoms, including posttraumatic stress disorder (PTSD; De Bellis and Kuchibhatla, 2006; Holmes et al., 2018; Baldaçara et al., 2011; Huggins et al., 2024; Huggins et al., 2026). These cross-sectional findings point to the cerebellum as a promising, yet understudied, neural substrate through which chronic adversity may confer risk for psychopathology.

Despite these emerging insights, few studies have investigated how exposure to chronic adversity - particularly neighborhood socioeconomic disadvantage and childhood maltreatment - jointly relate to cerebellar structure (Brooks et al., 2016; Peterson et al., 2024). Importantly, these forms of adversity are not necessarily correlated and may exert distinct effects on neurodevelopment, as neighborhood disadvantage reflects a chronic environmental context of structural deprivation, whereas childhood maltreatment involves direct, often interpersonal experiences of harm (McLaughlin et al., 2019; Wade et al., 2022). Although acute trauma exposure, childhood maltreatment, and neighborhood disadvantage represent distinct forms of adversity, they may partially overlap in their sources of stress. Specific dimensions of childhood trauma, for example deprivation, may further overlap with socioeconomic disadvantage (Maguire-Jack and Font, 2017). However, neighborhood-level disadvantage encompasses characteristics of the broader social and physical environment that are not captured by individual experiences (George et al., 2023; Huggins et al., 2022). Additionally, PTSD symptoms are further distinguished by their proximal relationship to a traumatic event, and prior adversity may contribute to vulnerability or resilience following such events (Breslau et al., 2014; Wong et al., 2023). Accordingly, the present study examined neighborhood disadvantage and childhood trauma as related, but conceptually distinct, forms of adversity in adults recently exposed to traumatic events, while separately evaluating PTSD symptom severity following the acute trauma. Examining both exposures within a trauma-exposed sample allows for a more nuanced understanding of whether adversity broadly relates to cerebellar morphology, or whether distinct patterns emerge across personal versus environmental forms of adversity. Addressing this gap is critical, given the cerebellum's integrative role in cognitive and emotional functioning and its extended developmental window of vulnerability.

The present study, therefore, examines associations between chronic adversity and cerebellar volume in a sample of adults recently exposed to trauma. Specifically, in a sample of trauma-exposed adults, we assessed the relationships between total cerebellar volume and (1) neighborhood disadvantage, indexed by the ADI, and (2) childhood maltreatment, measured with the Childhood Trauma Questionnaire (CTQ). Given emerging evidence suggesting regional specificity in cerebellar structure-function relationships (Stoodley and Schmahmann, 2018), we also conducted exploratory analyses examining associations with subregional cerebellar volumes. Finally, we explored whether total cerebellar volume was associated with future PTSD symptom severity, providing insight into potential pathways linking chronic adversity, cerebellar structure, and trauma-related psychopathology. We hypothesized that higher levels of both neighborhood disadvantage and childhood maltreatment would be associated with smaller cerebellar volumes, and that reduced cerebellar volume would relate to greater PTSD symptom severity.

## 2 Methods

### 2.1 Participants

Participants were from the Imaging Study of Trauma and Resilience (iSTAR) study, a longitudinal investigation of neurobiological predictors of PTSD in adults (Tomas et al., 2022; Webb et al., 2021a, Webb et al., 2021b; Bird et al., 2021). The participants were recruited from the emergency department of a Level 1 trauma center in southeastern Wisconsin after acute traumatic injury. Eligible participants (N = 220) were required to (1) speak English, (2) be between 18 and 65 years of age, (3) score ≥3 on the Predicting PTSD Questionnaire (Rothbaum et al., 2014), and (4) score ≥13 on the Glasgow Coma Scale (Sternbach, 2000). Exclusion criteria included moderate-to-severe traumatic brain injury, spinal cord injury, history of psychotic or manic symptoms, traumatic injuries resulting from suicide attempt or self-harm, or MRI contraindications (e.g., pregnancy).

Within 2-4 weeks post-trauma (T1), participants completed structural and functional MRI scans and a battery of self-report measures. At 6 months (T2), they completed follow-up assessments of depression, anxiety, and PTSD symptoms. For the present analyses, participants were included if they had useable cerebellar volume data at T1. Subsets of this sample also had valid data for neighborhood socioeconomic disadvantage (T1), childhood maltreatment (T1), and PTSD symptom severity (T2).

### 2.2 Measures

#### 2.2.1 Neighborhood socioeconomic disadvantage

Neighborhood disadvantage was measured using the ADI (Kind and Buckingham, 2018). Participants’ residential addresses were geocoded using the University of Wisconsin School of Medicine and Public Health Neighborhood Atlas (2018 ADI, v3.0; data downloaded February 2020). ADI scores comprise factor-based percentile rankings (1 = most advantaged; 100 = most disadvantaged) based on 17 census block-group variables from the 2014–2018 American Community Survey reflecting domains of income, education, housing quality, and employment.

#### 2.2.2 Childhood maltreatment

Childhood maltreatment was assessed using the Childhood Trauma Questionnaire-Short Form (CTQ-SF; Bernstein et al., 2003). The CTQ-SF is a 28-item retrospective self-report measure designed to screen for five distinct subtypes of maltreatment: emotional abuse, physical abuse, sexual abuse, emotional neglect, and physical neglect. Participants rate statements regarding their childhood experiences on a 5-point Likert scale ranging from 1 (*never true*) to 5 (*very often true*). The CTQ-SF has demonstrated robust psychometric properties, including measurement invariance and criterion validity across diverse clinical and community populations (Bernstein et al., 2003). A total CTQ-SF score was calculated by summing the 25 clinical items, yielding a range of 25 to 125. Higher scores indicate a greater severity of childhood maltreatment.

#### 2.2.3 PTSD symptom severity

The Clinician-Administered PTSD Scale for DSM-5 (CAPS-5; Weathers et al., 2018) was used to assess PTSD symptom severity at T2. This semi-structured interview evaluates the 20 DSM-5 symptom criteria for PTSD on a 0-4 scale. The CAPS-5 has demonstrated excellent psychometric properties and is considered the gold standard for PTSD diagnosis (Weathers et al., 2018). A cumulative severity score was generated by summing the 20 core symptom items, with higher scores reflecting greater symptom severity.

#### 2.2.4 MRI data acquisition and processing

Structural MRI scans were acquired on a 3T General Electric Discovery MR750 scanner with a 32-channel head coil. High-resolution T1-weighted spoiled gradient-echo (SPGR) anatomical images were obtained (FOV = 240 mm; matrix = 256 × 224; slice thickness = 1 mm; 150 slices; TR/TE = 8.2/3.2 ms; flip angle = 12°; voxel size = 0.94 × 1.07 × 1.00 mm). Intracranial volume (ICV) was derived using FreeSurfer v6.0 (https://surfer.nmr.mgh.harvard.edu/).

Cerebellar segmentation was conducted using the Automatic Cerebellum Anatomical Parcellation using U-Net with Locally Constrained Optimization (ACAPULCO) v0.2.1 algorithm (Han et al., 2020; see Fig. 1). ACAPULCO is a deep-learning-based method for automated cerebellar parcellation. Structural images underwent preprocessing that included N4 bias-field correction, Gaussian smoothing (SD = 3 mm), and normalization to MNI space. The ACAPULCO pipeline first localized the cerebellum by generating a three-dimensional bounding box and then segmented the structure into a total cerebellar volume and 28 anatomically defined subregions. These included anterior hemispheric lobules (I-V), posterior hemispheric regions (lobules VI-IX and crus I-II), the flocculonodular lobe (lobule X), vermal subdivisions, and the corpus medullare. Total cerebellar volumes were used in primary analyses, and additional exploratory analyses evaluated volumes of individual subregions.

**Fig. 1:**
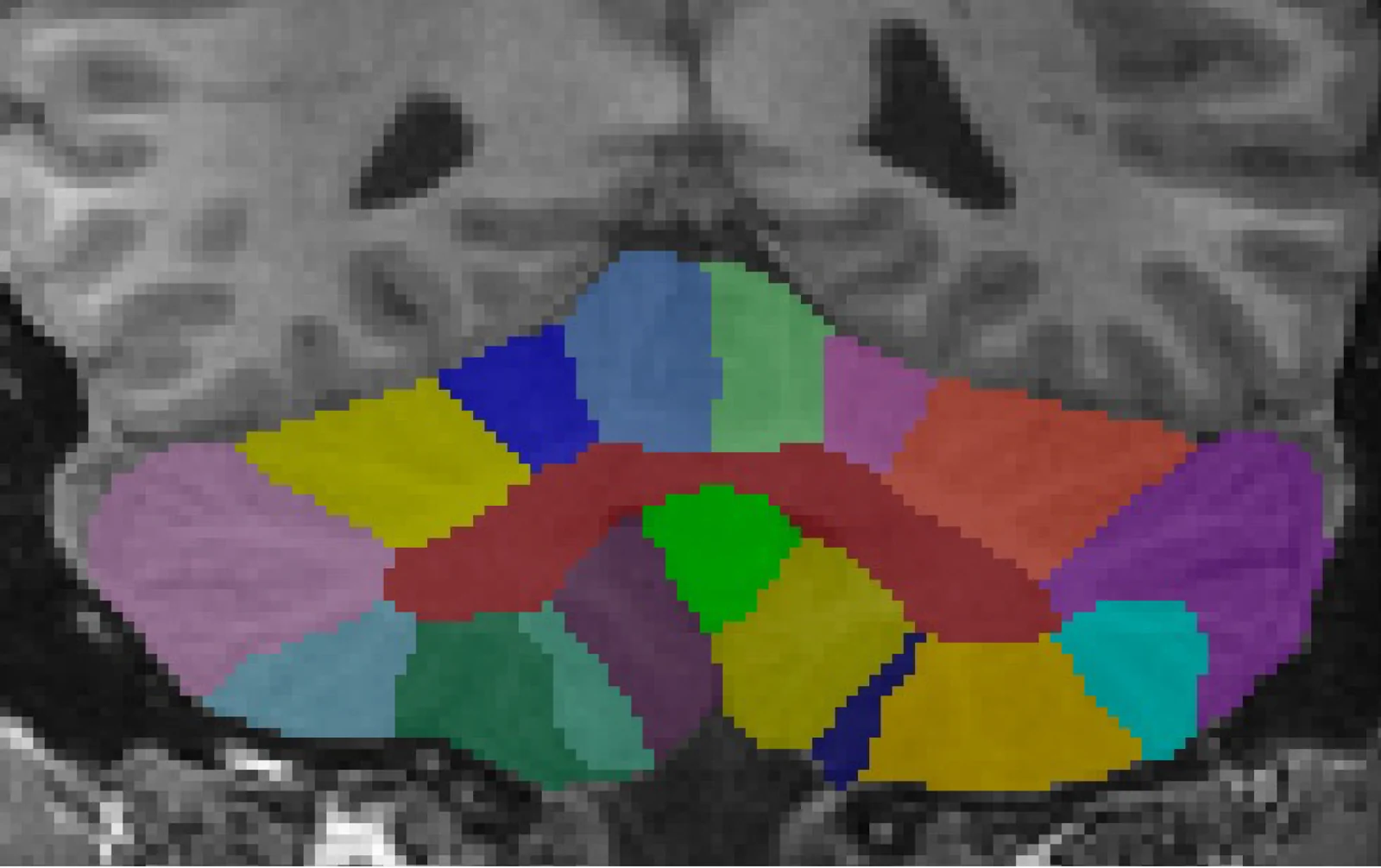
Example cerebellar segmentation output generated using ACAPULCO (Han et al., 2020).

Cerebellar segmentation outputs underwent quality control following the ENIGMA protocol (Kerestes et al., 2022), including visual inspection of raw images, statistical outlier detection of subregional volumes, and manual verification of flagged segmentations using overlay of the parcellation mask on native T1-weighted images.

### 2.3 Statistical analysis

Analyses were conducted in R (v4.3.2). Associations between adversity measures (ADI, total CTQ) and cerebellar volume were examined separately using multiple linear regression. Associations were also performed adjusting for age, gender, and ICV. Although neighborhood- and individual-level socioeconomic circumstances are closely related, prior research has shown that neighborhood disadvantage may have independent and compounding effects on health (Ross and Mirowsky, 2001). To account for the potential for overlapping constructs being captured by neighborhood- and individual-level socioeconomic metrics, sensitivity analyses additionally adjusted for income and educational attainment to examine whether associations with ADI persisted beyond individual-level socioeconomic characteristics (Gard et al., 2020; Webb et al., 2021a, Webb et al., 2021b; Huggins et al., 2022). Exploratory models tested whether total cerebellar volume mediated the relationship between adversity and PTSD symptom severity, and whether associations differed by cerebellar subregion. False discovery rate (FDR) correction was applied to account for multiple comparisons across the 28 cerebellar subregions. Supplementary analyses examined threat- and deprivation-related childhood maltreatment (Sheridan and McLaughlin, 2014) in relation to one another, neighborhood disadvantage, and total cerebellar volume.

The present analyses were not pre-registered. Analyses examining associations between neighborhood disadvantage and childhood maltreatment with total cerebellar volume were specified a priori, whereas subregional and mediation analyses were exploratory.

## 3 Results

### 3.1 Participants

The full sample consisted of 220 participants. Participants had a mean age of 33.28 years (*SD* = 10.94, range = 18-59 years), and 52.7% identified as female. Volumetric cerebellum data at T1 was available for 178 participants, and the mean total cerebellar volume was 132,225 mm^3^ (*SD* = 14,319 mm^3^). Neighborhood disadvantage, indexed using the ADI, was available for 152 participants. ADI scores in this sample demonstrated substantial variability with a skew towards greater disadvantage (mean = 68.62, *SD* = 21.58; range = 11-100). Childhood maltreatment scores were available for 159 participants (mean = 44, *SD* = 16.64; range = 25-107) and reflected a broad range of maltreatment exposure. Additional descriptive statistics are presented in Table 1. Bivariate correlations among study variables are presented in Table 2.

**Table 1:** Sample characteristics.

Characteristic	N = 220^a^
Gender	
Female	116 (52.7%)
Male	104 (47.3%)
Age (years)	33.28 (10.94)
Income	
$0–$10,000	44 (20.8%)
$10,000–$20,000	31 (14.6%)
$20,000–$30,000	37 (17.5%)
$30,000–$40,000	17 (8.0%)
$40,000–$50,000	18 (8.5%)
$50,000–$60,000	16 (7.5%)
$60,000–$70,000	13 (6.1%)
$70,000–$80,000	13 (6.1%)
$80,000–$90,000	3 (1.4%)
$90,000–$100,000	9 (4.2%)
Greater than $100,000	11 (5.2%)
Education (years)	13.50 (1.99)
Race	
Asian	4 (1.9%)
Black or African American	126 (61.2%)
More than one race	15 (7.3%)
White	61 (29.6%)
Ethnicity	
Hispanic or Latino	20 (9.2%)
Not Hispanic or Latino	197 (90.8%)
CAPS-5 Total (T2)	13.70 (12.05)
ADI	68.62 (21.58)
CTQ Total	44.01 (16.64)
Total Cerebellum Volume (mm^3^)	132,225.48 (14,319.06)
Intracranial Volume (ICV)	1,411,464.95 (158,973.37)

a Values are M (SD) for continuous variables and n (%) for categorical variables. Missing data: ADI = 68 cases, CTQ = 61 cases, Total Cerebellum Volume = 42 cases, ICV = 42 cases, CAPS-5 Total = 26 cases, Race = 14 cases, Income = 8 cases, Ethnicity = 3 cases. ADI, Area Deprivation Index; CTQ, Childhood Trauma Questionnaire.

**Table 2:** Correlations between variables of interest Means, standard deviations, and correlations with confidence intervals.

Variable	*M*	*SD*	1	2	3	4	5	6	7
1. Age	33.03	10.71							

2. ICV	1411464.95	158973.37	−0.14						
			[-0.28, 0.01]						

3. Total Cerebellum Volume	132225.48	14319.06	−0.29**	0.74**					
			[-0.42, −0.15]	[0.67, 0.80]					

4. ADI	68.17	21.59	0.03	−0.15	−0.29**				
			[-0.13, 0.19]	[-0.30, 0.01]	[-0.43, −0.14]				

5. CTQ Total	42.91	16.40	0.07	−0.12	−0.20*	0.13			
			[-0.09, 0.22]	[-0.27, 0.04]	[-0.34, −0.04]	[-0.03, 0.29]			

6. CAPS Total	13.57	12.22	−0.08	−0.05	0.04	0.07	0.27**		
			[-0.24, 0.08]	[-0.21, 0.11]	[-0.12, 0.20]	[-0.10, 0.24]	[0.11, 0.41]		

7. Income	4.28	3.11	−0.01	0.22**	0.25**	−0.47**	−0.25**	−0.17*	
			[-0.15, 0.14]	[0.08, 0.36]	[0.10, 0.38]	[-0.59, −0.34]	[-0.39, −0.09]	[-0.32, −0.01]	

8. Years of Education	13.55	2.06	0.08	0.03	0.05	−0.35**	−0.10	−0.08	0.43**
			[-0.07, 0.23]	[-0.12, 0.18]	[-0.10, 0.20]	[-0.48, −0.20]	[-0.25, 0.06]	[-0.24, 0.08]	[0.30, 0.55]

*Note. M* and *SD* are used to represent mean and standard deviation, respectively. Values in square brackets indicate the 95% confidence interval for each correlation. ADI, Area Deprivation Index; CTQ, Childhood Trauma Questionnaire, ICV, Intracranial Volume. *p < .05; **p < .01.

### 3.2 Associations between neighborhood disadvantage, childhood maltreatment, and total cerebellum volume

In unadjusted models, greater neighborhood disadvantage was significantly associated with smaller total cerebellar volume (*F*(1,150) = 14.09, adjusted *R*^2^ = 0.08, *b* = −186.34, *p* < .001). This association remained robust after adjusting for ICV, age, and gender (*F*(4,147) = 56.59, adjusted *R*^2^ = 0.60, *b* = −120.17, *p* < .001, partial *R*^2^ = 0.08). Greater total childhood maltreatment was also associated with smaller total cerebellar volume in an unadjusted model (*F*(1,157) = 6.49, adjusted *R*^2^ = 0.03, *b* = −177.04, *p* = .012). This association remained statistically significant after adjustment for ICV, age, and gender (*F*(4,154) = 56.35, adjusted *R*^2^ = 0.58, *b* = −94.00, *p* = .043, partial *R*^2^ = 0.03). These associations are illustrated in Fig. 2.

**Fig. 2:**
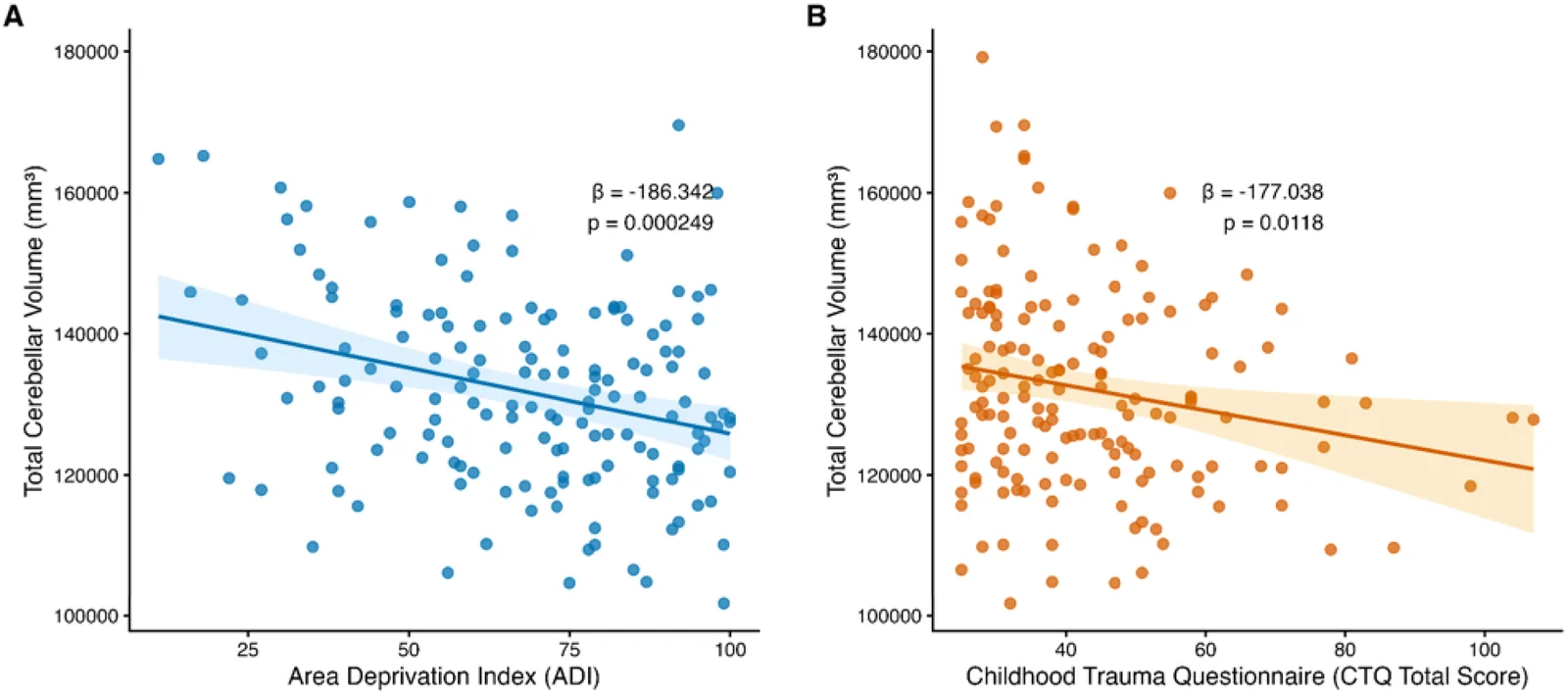
Associations between adversity and total cerebellar volume. (A) Scatterplot depicting the relationship between neighborhood deprivation, measured using the Area Deprivation Index (ADI), and total cerebellar volume. (B) Scatterplot depicting the relationship between childhood maltreatment severity, measured using the Childhood Trauma Questionnaire (CTQ), and total cerebellar volume. Solid lines represent linear regression fits with shaded 95% confidence intervals.

The association between neighborhood disadvantage and total cerebellar volume remained significant after further adjustment for socioeconomic covariates. Specifically, greater neighborhood disadvantage continued to be associated with smaller cerebellar volume when income was included as an additional covariate (*F*(5,146) = 45.06, adjusted *R*^2^ = 0.59, *b* = −112.86, *p* = .003), when years of education was included as an additional covariate (*F*(5,143) = 44.04, adjusted *R*^2^ = 0.59, *b* = −124.77, *p* < .001), and when both income and years of education were included simultaneously as additional covariates (*F*(6,142) = 36.51, adjusted *R*^2^ = 0.59, *b* = −118.93, *p* = .003).

When neighborhood disadvantage and total childhood maltreatment were included in the same model adjusting for ICV, age, and gender, greater neighborhood disadvantage remained significantly associated with smaller total cerebellar volume (*F*(5,132) = 42.88, adjusted *R*^2^ = 0.60, *b* = −129.94, *p* < .001), whereas childhood maltreatment did not (*b* = −76.40, *p* = .104).

### 3.3 Subregional cerebellar volumes and neighborhood disadvantage

In unadjusted linear regression models examining neighborhood socioeconomic disadvantage as the predictor, greater disadvantage was associated with smaller volumes across a broad set of cerebellar subregions. Significant negative associations (*p* < .05) were observed in bilateral anterior and posterior lobules, including Left V, Right I-III, Right IV, Right V, Left VI, bilateral VIIB, bilateral VIIIB, Right VIIIA, bilateral X, as well as Vermis VIII and IX. In addition, neighborhood disadvantage was negatively associated with volume of the corpus medullare. Effects were uniformly negative, indicating that higher neighborhood disadvantage corresponded to reduced cerebellar volume across these regions. After adjusting for age, gender, and ICV, several of these associations remained statistically significant, although the number of significant regions was reduced. Further, some adjusted associations did not survive FDR correction for multiple comparisons. See Table 3 and Fig. 3 for complete subregional results, including FDR-corrected findings from adjusted models.

**Table 3:** Exploratory subregional analyses.

Lobe	Subregion	ADI (b, FDR p)	CTQ (b, FDR p)
Anterior lobe	Left.I.III	−0.561, 0.540	−0.407, 0.712
	**Right.I.III**	**−2.42, 0.014**	−1.43, 0.377
	Left.IV	0.804, 0.792	−5.675, 0.377
	Right.IV	−3.442, 0.290	−2.953, 0.528
	**Left.V**	**−5.274, 0.014**	−2.271, 0.528
	**Right.V**	**−3.742, 0.043**	−2.676, 0.401
Posterior lobe	Left.Crus.I	−2.25, 0.792	−7.668, 0.528
	Right.Crus.I	−8.308, 0.387	−9.941, 0.528
	Left.Crus.II	−3.659, 0.540	−3.393, 0.692
	Right.Crus.II	−9.325, 0.108†	−7.728, 0.401
	Left.VI	−7.198, 0.253	−3.803, 0.692
	Right.VI	−0.496, 0.949	3.081, 0.699
	**Left.VIIB**	**−10.358, 0.016**	−13.248, 0.122†
	Right.VIIB	−5.357, 0.218	−6.252, 0.377
	Left.VIIIA	−1.334, 0.792	7.008, 0.377
	Right.VIIIA	−5.405, 0.218	−5.724, 0.377
	**Left.VIIIB**	**−11.598, < 0.001**	−5.746, 0.377
	**Right.VIIIB**	**−10.033, 0.013**	−7.316, 0.377
	Left.IX	−1.397, 0.764	0.51, 0.913
	Right.IX	−2.926, 0.387	0.584, 0.913
Flocculonodular lobe	Left.X	−0.64, 0.218	0.452, 0.528
	**Right.X**	**−1.309, 0.014**	−0.008, 0.988
Vermis	Vermis.VI	1.738, 0.166	−1.005, 0.584
	Vermis.VII	−0.241, 0.792	−0.449, 0.692
	**Vermis.VIII**	**−2.785, 0.046**	−2.087, 0.377
	Vermis.IX	−0.604, 0.368	−1.312, 0.377†
	Vermis.X	−0.011, 0.953	−0.362, 0.377
	**Corpus.Medullare**	**−22.038, 0.013**	−14.18, 0.377

Note. Values represent adjusted models (b, FDR-corrected p), which included intracranial volume (ICV), age, and gender as covariates. † indicates regions significant in adjusted models before correction for multiple comparisons at p < .05 but not after FDR correction. ADI, Area Deprivation Index; CTQ, Childhood Trauma Questionnaire.

**Fig. 3:**
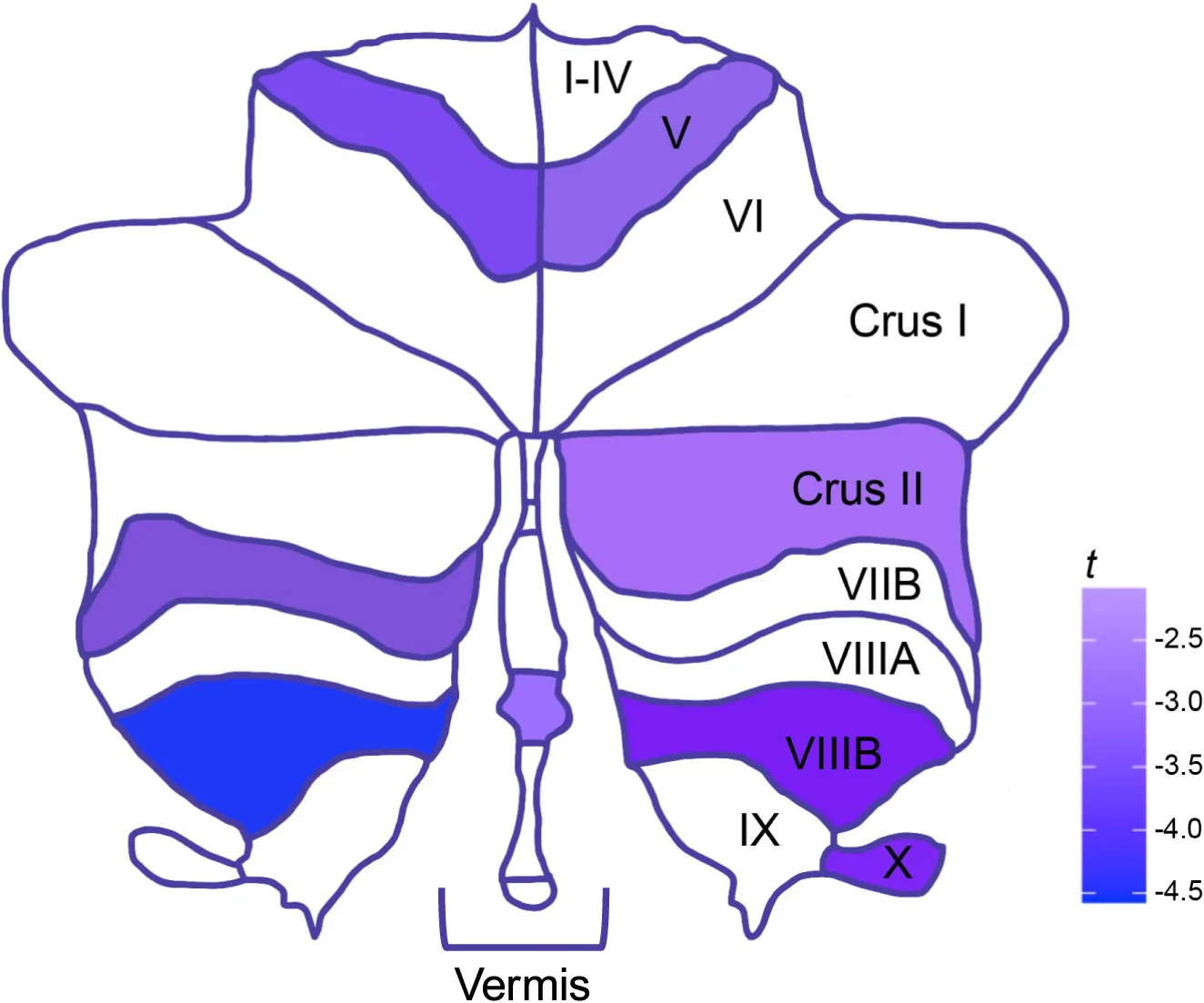
Significant effects of ADI on subregional cerebellar volumes. Test statistics from adjusted models, covarying for age, gender, and total intracranial volume are presented on a flattened cerebellum map based on the Spatially Unbiased Infratentorial (SUIT) atlas (Diedrichsen, 2006). Significant effects of ADI are plotted at uncorrected *p* < .05. The effect in right Crus II did not withstand correction for multiple comparisons (*p-FDR* = 0.108).

### 3.4 Subregional cerebellar volumes and childhood maltreatment

In unadjusted models, greater exposure to childhood maltreatment was associated with smaller volumes in a more limited set of cerebellar subregions relative to models examining neighborhood socioeconomic disadvantage. All significant effects were negative, indicating a relationship between lower regional cerebellar volumes and higher levels of childhood maltreatment. Following adjustment for age, gender, and ICV, only two of these associations remained statistically significant (Left VIIB, Vermis IX), indicating that most effects were substantially attenuated after accounting for covariates; moreover, no significant effects were retained following FDR correction for multiple comparisons. Complete results for all subregions from adjusted models are reported in Table 3.

### 3.5 Mediation analyses with PTSD symptom severity

We conducted mediation analyses to test whether total cerebellum volume mediated the associations between adversity (ADI and CTQ separately) and PTSD symptom severity post-trauma.

In unadjusted models, total cerebellum volume did not significantly mediate the relationship between ADI and PTSD symptoms (ACME = 0.007, 95% CI [−0.017, 0.033], *p* = .566), and neither the direct effect (ADE = 0.023, 95% CI [−0.079, 0.117], *p* = .638) nor the total effect (Total Effect = 0.030, 95% CI [−0.067, 0.121], *p* = .526) were significant. When covariates were included (age, gender, ICV), results were similar, with a non-significant mediation effect (ACME = −0.011, 95% CI [−0.038, 0.016], *p* = .408), non-significant direct effect (ADE = 0.034, 95% CI [−0.072, 0.132], *p* = .487), and non-significant total effect (Total Effect = 0.024, 95% CI [−0.074, 0.116], *p* = .597).

In unadjusted models of CTQ, cerebellar volume also did not mediate the relationship between CTQ and PTSD symptoms (ACME = 0.001, 95% CI [−0.020, 0.022], *p* = .881). The direct effect of CTQ on PTSD severity was significant (ADE = 0.193, 95% CI [0.028, 0.367], *p* = .020), as was the total effect (Total Effect = 0.194, 95% CI [0.032, 0.368], *p* = .017). In adjusted models, the mediation remained non-significant (ACME = −0.009, 95% CI [−0.033, 0.007], *p* = .268), while the direct effect of CTQ on PTSD remained significant (ADE = 0.192, 95% CI [0.030, 0.364], *p* = .017), as did the total effect (Total Effect = 0.182, 95% CI [0.022, 0.355], *p* = .022).

Overall, total cerebellum volume did not significantly mediate the effects of neighborhood disadvantage or childhood maltreatment on PTSD symptom severity. The results suggest that while childhood maltreatment shows a robust direct association with PTSD severity, the effect is not explained by total cerebellar volume in this sample.

### 3.6 Supplementary

Supplementary analyses revealed that neither dimension of childhood maltreatment (i.e., threat or deprivation; Liuzzi et al., 2023; Sheridan and McLaughlin, 2014) was significantly correlated with neighborhood disadvantage, and neighborhood disadvantage remained significantly associated with smaller total cerebellar volume when both maltreatment dimensions were included in the model (see Supplementary Materials and Table S1).

## 4 Discussion

The present study examined associations between chronic adversity and cerebellar structure in a sample of adults recently exposed to trauma. Specifically, our work focused on socioeconomic disadvantage and childhood maltreatment as distinct, yet related, forms of chronic adversity. Consistent with our hypotheses, greater neighborhood disadvantage was robustly associated with smaller total cerebellar volume. This relationship persisted after adjustment for ICV, age, and gender, as well as further controlling for individual-level socioeconomic factors, including income and education. Greater childhood maltreatment exposure was also associated with smaller total cerebellar volume in both unadjusted and adjusted models. Together, these findings highlight the cerebellum as a potential neural substrate through which both early and chronic adversity exposure may be linked to alterations in adulthood.

### 4.1 Neighborhood disadvantage, childhood maltreatment, and total cerebellar structure

Greater exposure to childhood maltreatment was associated with smaller total cerebellar volume, consistent with prior studies linking early adversity to cerebellar abnormalities in pediatric and adult populations, as well as animal studies. A systematic review by Mundorf et al. (2024) found that, in healthy populations, childhood maltreatment was linked to bilateral reductions in cerebellar volume and diminished functional connectivity both within the cerebellum and between cerebellar and frontal regions. Children and adolescents with documented reports of childhood maltreatment have been found to have reductions in cerebellar volume (Hanson et al., 2010; Edmisto et al., 2011; Lim et al., 2018). Parallel evidence from animal studies indicated that early adversity can induce cerebellar structural and molecular changes, including shifts in stress hormone receptor levels, cell density, and neuroinflammatory markers, even in the absence of observable behavioral pathology (Banerjee et al., 2011; Moussa-Tooks et al., 2020; Wilber et al., 2007). Together, these findings underscore the cerebellum as a consistent target of early adversity across species, independent of clinical outcomes.

The observed association between greater neighborhood disadvantage and smaller total cerebellar volume extends prior work linking neighborhood socioeconomic conditions to neural structure and function. Previous studies have demonstrated that neighborhood socioeconomic disadvantage is associated with alterations in brain regions involved in threat processing, cognitive control, and emotion regulation, including the prefrontal cortex and hippocampus (Taylor et al., 2020; Webb et al., 2021a, Webb et al., 2021b). Our results suggest that the cerebellum may represent an additional, and relatively underexplored, neural substrate through which chronic exposure to disadvantaged environments exerts neurobiological effects. Importantly, the association between neighborhood disadvantage and cerebellar volume in the present study remained significant even *after* additional adjustment for individual-level socioeconomic factors, including income and education. While neighborhood disadvantage and individual socioeconomic position may often be related, neighborhood-level measures might capture broader contextual exposures with neurobiological consequences that are not fully accounted for by individual socioeconomic statuses (Gard et al., 2020). This pattern suggests that the relationship between neighborhood disadvantage and cerebellar structure goes beyond individual socioeconomic status. For example, neighborhood disadvantage may capture additional contextual stressors, such as environmental instability, exposure to violence, limited access to resources, and chronic uncertainty, that increase cumulative stress beyond personal socioeconomic circumstances. These contextual exposures may accumulate across development and adulthood, contributing to long-term alterations in brain structure.

The cerebellum's protracted developmental trajectory may render it particularly sensitive to such cumulative environmental influences. Cerebellar maturation extends into adolescence and early adulthood, with ongoing synaptic pruning and myelination during periods when individuals may be differentially exposed to neighborhood-level stressors (Beckinghausen and Sillitoe, 2019). Chronic activation of stress-related biological systems, including glucocorticoid and inflammatory pathways, has been proposed as one mechanism through which socioeconomic adversity influences brain development (Tian et al., 2021). These processes may plausibly impact cerebellar structure.

### 4.2 Neighborhood disadvantage, childhood maltreatment, and cerebellar subregions

In exploratory subregional analyses, neighborhood disadvantage, rather than childhood maltreatment, demonstrated the most consistent and robust relationships with reduced volumes across the cerebellum. Even after covariate adjustment, greater neighborhood disadvantage was associated with smaller volumes in multiple cerebellar lobules spanning anterior (e.g., lobule V), posterior (e.g., VIIB, VIIIB), and flocculonodular (lobule X) regions, as well as the corpus medullare. The involvement of posterior lobules is particularly notable given their established roles in higher-order cognitive and affective processes, suggesting a possible structural pathway linking neighborhood-level adversity to downstream cognitive and emotional functioning (Moore et al., 2017).

By contrast, childhood maltreatment was associated with a narrower pattern of subregional cerebellar volume differences. While unadjusted models identified several regions of significantly smaller volumes within the posterior lobe and vermis, most associations did not survive adjustment for demographic factors and ICV. Moreover, no associations between subregional cerebellar volumes and childhood maltreatment withstood stringent correction for multiple comparisons. Still, the persistence of uncorrected associations in Left VIIB and Vermis IX after adjustment may indicate heightened vulnerability of these regions to childhood maltreatment. The cerebellar vermis, in particular, has been implicated in affective regulation and stress responsivity, and structural alterations in this region have been reported in prior studies of trauma-related psychopathology (Baldacara et al., 2011).

The differential patterns observed for neighborhood disadvantage and childhood maltreatment may reflect distinct mechanisms through which chronic adversity impacts brain structure. Here, it is notable that when childhood maltreatment and ADI were included in the same model, only neighborhood disadvantage emerged as a significant predictor of cerebellar volumes. Neighborhood disadvantage likely captures ongoing, cumulative environmental stressors that extend into adulthood, whereas childhood maltreatment represents earlier, temporally bounded exposures whose structural correlates may overlap with broader, whole-brain neurodevelopmental variation. Alternatively, the attenuation of childhood maltreatment-related effects after covariate adjustment may suggest that some associations operate through more global neurodevelopmental pathways rather than region-specific cerebellar alterations.

## 5 Implications for psychopathology risk

Although the present study focused primarily on structural associations, the findings have potential implications for understanding pathways linking chronic adversity to psychopathology risk. The cerebellum is increasingly recognized as a key contributor to cognitive and affective processes, including emotional regulation, threat learning, and executive control (Moreno-Rius, 2018; Borges et al., 2022; Stoodley and Schmahmann, 2010). Alterations in cerebellar structure have been documented in individuals with PTSD and other stress-related disorders, and smaller cerebellar volumes have been associated with greater symptom severity (De Bellis and Kuchibhatla, 2006; Huggins et al., 2024; Huggins et al., 2026; Blithikioti et al., 2022; Holmes et al., 2018). Prior work in the same sample of traumatically injured adults has examined traditionally studied cerebral regions, including hippocampal structure, in relation to neighborhood disadvantage and posttraumatic outcomes (Webb et al., 2021; Webb et al., 2021a, Webb et al., 2021b). The present findings extend this literature by identifying associations between chronic adversity and the cerebellum, a comparatively understudied region in this context.

In the current study, cerebellar volumes did not mediate associations between neighborhood disadvantage/childhood trauma and future PTSD symptom severity. Thus, while cerebellar structure may be sensitive to chronic adversity, variation in cerebellar structure alone may not explain the link between adversity exposure and risk for psychopathology, namely PTSD, in this sample. It is worth noting that the present sample was relatively small and consisted of community participants recruited following recent trauma exposure rather than a clinical PTSD sample, although PTSD symptom severity at 6-month follow-up was generally consistent with the broader literature (Breslau, 2009). As such, cerebellar volume may represent only one of multiple neurobiological pathways linking adversity exposure to subsequent post-trauma psychopathology risk.

Despite the absence of significant mediation effects, the observed associations between chronic adversity and cerebellar structure warrant further investigation into the potential relevance of cerebellar structure to posttraumatic outcomes. Future studies integrating longitudinal neuroimaging with repeated assessments of stress exposure and posttraumatic psychiatric symptoms will be essential for testing these relationships over time and clarifying their directionality.

## 6 Limitations and future directions

Several limitations should be acknowledged. First, the cross-sectional nature of the neuroimaging data precludes causal inferences regarding the directionality of associations between adversity and cerebellar volume. Second, childhood maltreatment was assessed retrospectively, which may be subject to recall bias. Third, neighborhood disadvantage was measured using residential address at a single time point, which may not fully capture cumulative neighborhood exposure across development. Additionally, sample sizes varied across analyses due to missing data on neighborhood and socioeconomic variables, which may have reduced statistical power in some models. Finally, the present sample consisted of adults recruited following acute traumatic injury, which should be considered when interpreting the generalizability of these findings. Prior trauma and adversity exposure may shape psychopathological and neurobiological responses to subsequent traumatic events, although these relationships appear to vary according to various characteristics and contexts around the trauma exposure (Ozer et al., 2003). Thus, associations between chronic adversity and cerebellar structure observed in the present trauma-exposed sample may not generalize to individuals without recent trauma exposure or to populations exposed to other types of traumatic events.

Despite these limitations, the study has several strengths, including the use of a well-characterized trauma-exposed sample, validated measures of neighborhood disadvantage and childhood maltreatment, and rigorous cerebellar segmentation methods. Future work should consider the interactive effects of multiple forms of adversity across the lifespan. Models that integrate these and other forms of chronic early life and adult stress exposure may provide a more comprehensive understanding of how chronic adversity shapes cerebellar development and function. Future studies should also examine the functional and psychological correlates of adversity-related differences in cerebellar structure. Integrating cerebellar morphometry with measures of cognitive performance, emotion regulation, and affective symptoms may help clarify the behavioral and clinical significance of these structural differences. Multimodal studies incorporating functional neuroimaging might further demonstrate whether structural differences are accompanied by alterations in cerebellar function or connectivity. Incorporating biological markers of stress, such as inflammatory or neuroendocrine measures, may additionally elucidate underlying mechanisms.

## 7 Conclusions

In summary, the present findings demonstrate that greater neighborhood socioeconomic disadvantage and greater childhood maltreatment are individually and robustly associated with smaller total cerebellar volume in adults recently exposed to trauma, independent of individual-level socioeconomic factors. These results highlight the cerebellum as a potentially important neural correlate of chronic adversity, particularly neighborhood disadvantage. Although the present findings did not provide evidence that cerebellar volume mediates associations between chronic adversity and PTSD symptoms, understanding the relationship between chronic adversity and cerebellar structure may help inform future research examining the neurobiological consequences of chronic socioeconomic and early life stress and their potential relevance to posttraumatic functioning.

## CRediT authorship contribution statement

**Jamie Terner:** Writing – original draft, Visualization, Formal analysis, Data curation, Conceptualization. **E. Kate Webb:** Writing – review & editing, Investigation. **Terri A. deRoon-Cassini:** Writing – review & editing, Investigation, Funding acquisition. **Christine L. Larson:** Writing – review & editing, Investigation, Funding acquisition. **Ashley A. Huggins:** Methodology, Conceptualization, Investigation, Writing – review & editing, Visualization.

## Ethics declaration

Written informed consent to take part in the study and to publish the article has been obtained from all participants or their legal representatives. The privacy rights of participants have been observed.

This study was performed in compliance with relevant laws, regulatory frameworks and guidelines where the research took place. This study was approved by the Institutional Review Board at the Medical College of Wisconsin.

## Funding

This work was supported by grant R01MH106574 from the National Institute of Mental Health.

## Declaration of competing interest

The authors declare that they have no known competing financial interests or personal relationships that could have appeared to influence the work reported in this paper.

## Data Availability

Data will be made available on request.
