# Supplementary material for: Chronic adversity and cerebellar structure: Links with neighborhood disadvantage and childhood maltreatment in adult trauma survivors
Source: Neurobiol Stress. 2026 Nov 2;45:100861. doi: 10.1016/j.ynstr.2026.100861 (PMC13769229; doi:10.1016/j.ynstr.2026.100861)
Supplement: Supplementary file 1 [file mmc1.docx]

**Supplementary Analyses: Threat- and Deprivation-Related Childhood Maltreatment**

**Methods**

Consistent with dimensional models of childhood maltreatment (Sheridan & McLaughlin, 2014), CTQ emotional abuse, physical abuse, and sexual abuse subscales were summed to create a CTQ threat-related variable, while emotional neglect and physical neglect subscales were summed to create a CTQ deprivation-related variable (Liuzzi et al., 2024). Supplementary analyses examined correlations among threat-related childhood maltreatment, deprivation-related childhood maltreatment, and neighborhood disadvantage. Linear regression models then examined associations between the childhood maltreatment dimensions and total cerebellar volume. Consistent with the primary analyses, all regression models adjusted for intracranial volume, age, and gender. Threat- and deprivation-related maltreatment were first examined separately (Models 1-2), followed by a model including both dimensions simultaneously (Model 3). A final model additionally included neighborhood disadvantage to examine the unique associations of neighborhood disadvantage and both childhood maltreatment dimensions with total cerebellar volume (Model 4).

**Results**

Threat-related maltreatment scores had a mean of 24.14 (*SD* = 11.24), and deprivation-related maltreatment scores had a mean of 18.93 (*SD* = 7.94). Neighborhood disadvantage was not significantly correlated with either threat-related maltreatment (*r* = .12, 95% CI [-.05, .28], *p* = .168) nor deprivation-related maltreatment (*r* = .11, 95% CI [-.06, .27], *p* = .213). Threat- and deprivation-related maltreatment were moderately positively correlated (*r* = .46, 95% CI [.33, .57], *p* < .001).

In adjusted models examining the childhood maltreatment dimensions separately, threat-related maltreatment was not significantly associated with total cerebellar volume (b = -110.25, p = .112), whereas greater deprivation-related maltreatment was associated with smaller total cerebellar volume (b = -205.90, p = .030). When threat- and deprivation-related maltreatment were entered simultaneously, neither threat (b = -48.66, p = .537) nor deprivation (b = -173.32, p = .110) was independently associated with total cerebellar volume. In the final model additionally including neighborhood disadvantage, greater neighborhood disadvantage remained significantly associated with smaller total cerebellar volume (b = -129.89, p < .001), whereas neither threat-related (b = -24.58, p = .754) nor deprivation-related maltreatment (b = -155.94, p = .148) was significantly associated with total cerebellar volume.

**Table S1. Supplementary Table**

|  | **Model 1: Threat**  **(N=160)** | **Model 2: Deprivation**  **(N=160)** | **Model 3: Both Dimensions**  **(N=160)** | **Model 4: Both Dimensions + ADI**  **(N=138)** |
| --- | --- | --- | --- | --- |
| Neighborhood disadvantage | --- | --- | --- | -129.89, p = <0.001*** |
| Threat-related maltreatment | -110.25, p = 0.112 | --- | -48.66, p = 0.537 | -24.58, p = 0.754 |
| Deprivation-related maltreatment | --- | -205.90, p = 0.030* | -173.32, p = 0.110 | -155.94, p = 0.148 |
| Intracranial volume | 0.07, p = <0.001*** | 0.07, p = <0.001*** | 0.07, p = <0.001*** | 0.06, p = <0.001*** |
| Age | -275.54, p = <0.001*** | -291.64, p = <0.001*** | -285.48, p = <0.001*** | -298.71, p = <0.001*** |
| Gender (male) | -805.40, p = 0.698 | -178.88, p = 0.930 | -391.21, p = 0.851 | 1306.87, p = 0.540 |
|  |  |  |  |  |

p < 0.05*, p < 0.01**, p < 0.001***
